# Access to ear and hearing care services in Cambodia: a qualitative enquiry into experiences of key informants

**DOI:** 10.1017/S0022215122002158

**Published:** 2024-01

**Authors:** C J Waterworth, M Marella, M F Bhutta, R Dowell, K Khim, P L Annear

**Affiliations:** 1 Department of Audiology and Speech Pathology, University of Melbourne, Australia; 2 Nossal Institute for Global Health, University of Melbourne, Australia; 3 Clinical and Experimental Medicine, Brighton & Sussex Medical School, Brighton, UK; 4 Department of ENT, Brighton and Sussex University Hospitals NHS Trust, Brighton, UK; 5 Monitoring, Evaluation and Learning, Access Program, Phnom Penh, Cambodia

**Keywords:** Hearing Loss, Otolaryngology, Health Services Accessibility, Health Policy, Cambodia

## Abstract

**Objective:**

In Cambodia, little is known about the state of ear and hearing care, or the roles providers or key stakeholders play in delivering services.

**Method:**

This was an exploratory study using semi-structured qualitative interviews and a questionnaire addressed to key stakeholders to explore their perceptions and experiences in providing services to people suffering from ear disease or hearing loss in Cambodia.

**Results:**

Several challenges were described including a lack of hearing services to meet the demand, especially outside Phnom Penh in primary care and aural rehabilitation. Supply-side challenges include a shortage of trained professionals, facilities and resources, poor co-ordination between providers, unclear referral pathways, and long wait times.

**Conclusion:**

Now is an opportune time to build on the positive trend in providing integrated care for non-communicable diseases in Cambodia, through the integration of effective ear and hearing care into primary care and strengthening the package of activities delivered at government facilities.

## Introduction

Hearing loss is the most frequent sensory impairment,^1^ with an estimated impact of over 1.57 billion people globally experiencing a mild or worse hearing loss, of which 80 per cent occurs within low- and middle-income countries.^2,3^ Despite the growing burden, access to ear and hearing care in low- and middle-income countries remains disproportionately low, patterned by a range of socio-cultural, demographic and structural challenges. In Cambodia, access to ear and hearing care is available through the public, private and non-governmental organisation sectors, but it is limited by the significant shortage of providers, location of facilities, paucity of equipment and resources, and a lack of policy implementation for hearing-related disability and rehabilitation.^4^ In the public sector, there are nine hospitals with ENT departments across the country, with five situated in the capital. In the private sector, two major private hospitals have ENT departments, a few polyclinics have visiting ENT specialists, and to our knowledge, there are only two private hearing aid providers. The non-governmental organisation sector consists of several organisations with international links that provide diagnostic audiology, aural rehabilitation and specialty surgical treatment of chronic ear disease.

Primary healthcare can be an effective tool in achieving universal health coverage, reducing the burden on higher-level health facilities.^5^ Health system strengthening through integrating ear and hearing care into the primary healthcare framework, making use of task-sharing approaches for community health workers in hearing loss identification, prevention and rehabilitation, has been raised as a critical element in delivering cost-effective interventions into mainstream health services.^6,7^ Integration of care into primary healthcare is, however, a complex strategy involving numerous organisations, stakeholders, providers and interventions, and detailed integration strategies remain unclear.^8^ The World Health Organization (WHO) has set an ambitious goal that ‘clinical ear and hearing care services must be accessible, integrated within national health services and delivered across all levels of care’.^3^

In Cambodia, little is known on the state of ear and hearing care service provision, or the roles that providers or key stakeholders might play in integrating ear and hearing care services into primary healthcare. This study aims to understand the perceptions and experiences of service providers and stakeholders (key informants) in providing services to people suffering ear disease or hearing loss in Cambodia. We anticipate the findings of this study will contribute to a better understanding of ear and hearing care practices, informing public policies and programmes that support the provision of services that address health disparities and improve health outcomes of those suffering from ear or hearing conditions.

### Background

In 2017, the World Health Assembly unanimously adopted a resolution for the prevention of deafness and hearing loss, calling on member states to ensure ear and hearing care is accessible to people across their lifespan.^9^ Multiple barriers exist, however, to bring ear and hearing care under the banner of universal health coverage, with significant variations in service delivery capacity in low- and middle-income countries.^10^ Within low- and middle-income countries, there is a critical shortage of services and trained professionals in ear and hearing care, including otolaryngologists, audiologists, speech and language therapists, and teachers of the deaf,^11^ which means marginalised and socially disadvantaged populations often cannot access the care they need.

The government of Cambodia is committed to working towards attaining the Sustainable Development Goals, is a signatory of the United Nations Convention on the Rights of Persons with Disabilities^12^ and is implementing national policy to support disability-inclusion through the Incheon strategy for Asia Pacific.^13,14^

In 1996, the Cambodian public health system introduced nominal user fees at government health facilities with the aim of increasing the quality and coverage of health services.^15^ Several successful social health programmes were initiated, including the health equity fund and voluntary community-based health insurance and private insurance schemes in order to reduce out-of-pocket costs.^16^ Public healthcare centres deliver basic curative and preventative care through a minimum package of activities within 81 operational (health) districts, which 24 provincial health departments govern.^17^ Several operational districts usually make up a province, and each has a referral hospital delivering a complementary package of activities. Referral hospitals provide primary care through to more complex healthcare services, including surgical care, and orchestrate the movement of patients between health centres and national hospitals.^18,19^ Treatment targeting major non-communicable diseases has been successfully integrated into the Cambodian public health system.^20^ For example, the last 30 years have seen a reduction in maternal mortality,^21^ supported by investment in transport infrastructure and facilitation of trained midwives into remote health centres,^22^ and increased access to mental health, diabetes and hypertension services through better intergration into primary healthcare services.^23,24^ However, recent reports indicate that despite this investment and progress, the vast majority of the population, especially those in rural areas, are still unable to access quality healthcare services.^25,26^

In 2016, most ear and hearing care services were provided by non-governmental organisations, primarily in Phnom Penh.^27^ All Ears Cambodia has five clinics across the country and works in partnership with over 70 aid organisations and 5 public hospitals providing preventative education, low-cost hearing aids and primary healthcare.^28,29^ The Children's Surgical Centre in Kien Khleang was one of the first hospitals to employ Cambodian surgeons able to perform tympanoplasty and mastoidectomy.^30^ Impact Cambodia, an international non-governmental organisation, has executed several ear and hearing care projects, including outreach and prevention programmes in collaboration with implementing partners.^31^ Alongside the non-governmental organisations, there are a growing number of government hospitals with ENT Departments in Phnom Penh, including Preah Ang Duong Hospital, the Khmer-Soviet Friendship Hospital, Calmette Hospital, National Paediatric Hospital and Preah Kossamak Hospital. In 2019, a cochlear implant programme began at Preah Ang Duong Hospital and National Paediatric Hospital, where recipients pay for implantation and early intervention, including speech therapy. There are also a few scattered private clinics offering ear and hearing care rehabilitation services, and private hospitals with ENT departments now include the Royal Phnom Penh and Sunrise hospitals. To our knowledge, there are only four hospitals outside Phnom Penh with ENT departments: the Provincial Battambang Referral Hospital, Provincial Preah Vihear Referral Hospital, Kampong Chhnang Referral Hospital and the Chea Chumneas Referral Hospital in Kandal province.

Two disability organisations operate in Cambodia, serving adults and children with significant hearing loss. Special education for children with hearing impairment (from preparatory school to year 12) is provided by Krousar Thmey, under the Ministry of Education, Youth and Sports, with five schools located throughout the country.^25^ Most children at the school have hearing devices and have been supported by international visiting organisations, including Enfants Sourds du Cambodge, a non-governmental organisation based in France.^32^ Adults with severe to profound hearing loss and limited verbal communication can attend the Maryknoll Deaf Development Programme in Phnom Penh, where they learn Cambodian sign language and engage in vocational training.^33^ However, the overwhelming majority of the estimated 61 000 deaf children and adults throughout Cambodia do not have access to sign language^34^ or to educational or vocational support.

## Materials and methods

To gain an understanding of the current state of play on provision and uptake of ear and hearing care services in Cambodia, we undertook a qualitative, exploratory and descriptive approach to data acquisition and analysis, using semi-structured interviews and a questionnaire of key informants.

### Analytical framework

**Fig. 1.**
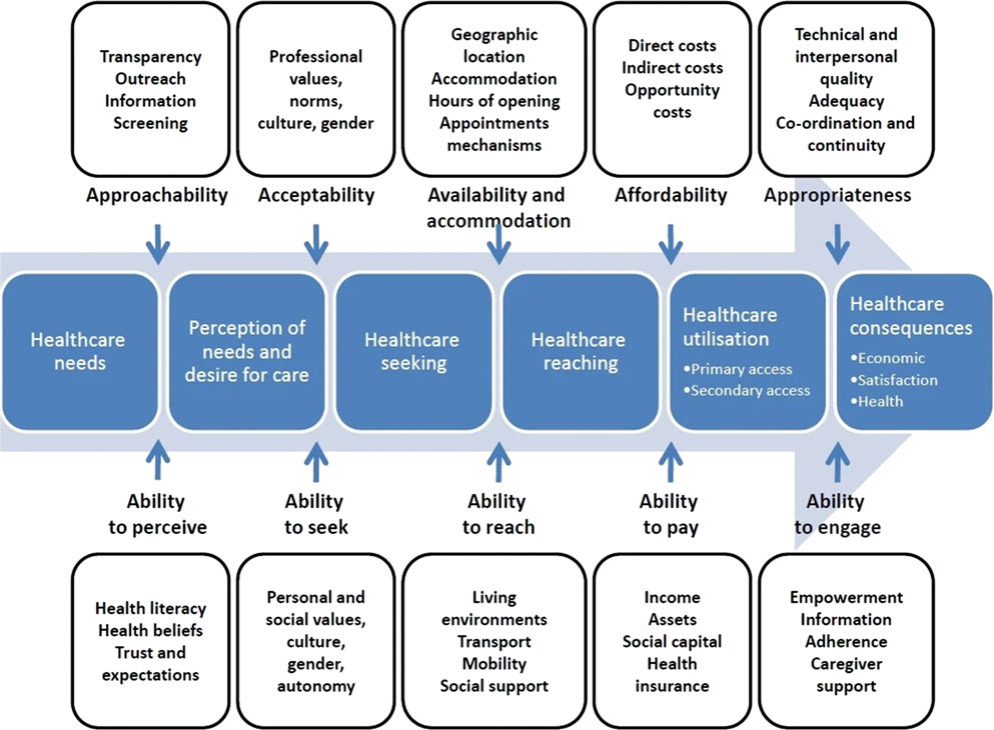
Conceptual framework of access to healthcare by Levesque *et al*.^35^

In order to evaluate access, we employed Levesque's conceptual framework, which has gained increasing recognition as a foundational model in the health service literature (Figure 1).^35^ This framework builds on previous conceptualisations of access^36^ and considers five key dimensions along the healthcare-seeking journey that capture supply- and demand-side determinants of access.

Supply-side determinants include: approachability (indicates people facing healthcare needs can identify the service exists, can be reached and can impact their health outcome), acceptability (refers to cultural and social factors that determine a users’ likelihood to accept aspects of a health service), availability (indicates the adequacy of supply and resource factors determining how services can be reached in a timely manner and have sufficient capacity to provide services with adequate resources), affordability (reflects the capacity of individuals to spend their resources and time to use services) and appropriateness (refers to the fit between a service and the users’ needs, such as quality of health services, integration and continuity of care).

Demand-side determinants refer to the user's capacity to perceive, seek, reach, pay for and engage with the healthcare service. In studying the accessibility of ear and hearing care services in Cambodia, we draw upon this foundational model allowing operationalisation of access along the pathway of utilisation of ear and hearing care, from the perception of need to the outcomes of service use.

### Recruitment of participants

We approached key informants currently working in the field of ear and hearing care in Cambodia through mutual connections and conducted in-depth interviews. As the public and non-governmental organisation sectors cover the majority of ear and hearing care services, we ensured the majority of interviewees represented those sectors. A purposive sampling strategy was employed to target 15 ear and hearing care professionals, representing expertise from the public (*n =* 5), non-governmental organisations (*n =* 8) and special education sectors (*n =* 2). We approached two providers from the private sector, but they did not wish to participate. Most providers we interviewed worked with children (*n =* 13). Participant characteristics are described in Table 1. Participants primarily provided services in ENT (*n =* 6), audiology (*n =* 5), other medical healthcare (*n =* 2) and special education for the hearing impaired (*n =* 2). Several providers held dual positions in public, private or non-governmental organisation hospitals (*n =* 4). The majority of participants were male (10 of 15 = 67 per cent). Twelve participants agreed to participate in the semi-structured interviews and three in the questionnaire.

**Table 1.** Participant demographic information

Participant	Sex	Location of work	Clinical service	Service management	Advocacy & support	Position, primary facility
01	F	Urban	X			Provider, NGO
02	M	Urban		X		Director, NGO
03	M	Urban/rural		X	X	Teacher, special education
04	M	Urban/rural	X			Other medical provider, NGO
05	F	Urban	X			Provider, public hospital
06	F	Urban/rural	X			Provider, NGO
07	F	Urban	X			Provider, public hospital
08	M	Urban/rural	X	X	X	Director, disability organisation special education
09	F	Rural	X			Other medical provider, NGO
10	M	Rural	X			Other medical provider, NGO
11	M	Urban	X			Provider, public hospital
12	M	Urban/rural	X		X	Director, NGO
13 (questionnaire)	M	Urban	X	X	X	Director, NGO
14 (questionnaire)	M	Urban/rural	X			Provider, public hospital
15 (questionnaire)	M	Urban/rural	X			Provider, public hospital

F = female; M = male; NGO = non-governmental organisation

### Interview approach and data analysis

Participants were invited to participate via e-mail or telephone. Data collection was conducted between March 2020 and March 2022 either in person (*n =* 7), online (*n =* 5) or through an online questionnaire (*n =* 3). The interview guide and questionnaire included a number of open-ended questions (see questionnaire and topic guide in the supplementary material, available on *The Journal of Laryngology & Otology* website). The initial discussion was aimed toward their professional background to ensure interview questions were appropriately framed. The interviews focused on perceptions of the need for their service, utilisation, satisfaction and acceptance of their service in the community, followed by perceptions of the structural, financial or socio-cultural barriers to ear and hearing care service provision and uptake. Towards the end of the interview, participants were invited to share their thoughts on potential strategies to improve access to ear and hearing care. Interviews were piloted to refine the interview guide. Key informants were referred to under the general term of ‘providers of ear care’ to ensure anonymity when presenting quotations.

Interviews were conducted in English (with assistance from a Khmer interpreter for one interview), with an average duration of about one hour. Interviews were digitally audio-recorded, transcribed verbatim (excluding Khmer), content coded and analysed using nVIVO qualitative data analysis software (version 12; QSR International, Melbourne, Australia). Transcripts were anonymised with names and other identifying information omitted. Codes were inductively derived from interview and questionnaire responses, compiled into categories and merged into main determinants according to the dimensions of the Levesque framework.^35^ Poignant quotations were selected to evidence key findings.

Initial findings, themes and potential solutions were presented back to a focus group of study participants for further clarification, interpretation and development. Discussion included evaluation of the recommended solutions regarding their applicability and impact on improving access.

### Rigor and trustworthiness

We undertook several techniques to ensure rigor and trustworthiness: selection of participants using maximum variation sampling (transferability), member checking to provide participants opportunity to review and correct interview transcripts (confirmability), concurrent data collection and analysis (dependability), peer debriefing of recommendations (credibility), and representation of citations from most participants (authenticity).

## Results

Stakeholders described several overarching supply and demand-side themes (Table 2), as well as potential strategies to mitigate some of these challenges (Table 3). We provide example quotations and excerpts from interviews to provide additional context.

**Table 2.** Summary of qualitative findings

Theme	Detailed findings	
	Supply	Demand
Approachability	Low prioritisation of EHC within the health system with no clear national plan. Ineffective marketing & public health campaigns	Low awareness of hearing loss, causes, interventions or preventative measures in society. Lack of perceived seriousness of the problem at the government level. Acceptance of traditional beliefs
Acceptability	Low government awareness of the social impact of hearing loss or deafness. Lack of consensus on the method of Khmer sign language	Stigma & discrimination in Khmer society toward hearing loss & use of hearing aids. ‘Healthcare shopping’ because of lack of trust in health system. Family influence & priority setting. Fear of doctors, surgery or anaesthesia
Availability	Lack of trained EHC personnel, equipment, instruments & facilities, especially outside Phnom Penh. Lack of representation of EHC services in primary level healthcare facilities. Lack of affordable quality hearing devices, inadequate supply chain & no local representation from device manufacturers. Health services at capacity. Lack of EHC training opportunities & reliance on international volunteers	Poor transport infrastructure. Geographical distance from care
Affordability	Direct & indirect costs of equipment & resources (e.g. hearing aids or surgical equipment). Opportunity costs for seeking treatment	Lack of funding mechanisms for EHC, hearing aids, cochlear implants or assistive listening devices
Appropriateness	Segregated facilities that rarely cross refer. Lack of infrastructure & co-ordination of healthcare, especially in rural or remote areas. Unregulated pharmaceutical industry. Low investment from government in EHC	Lack of belief in medical processes

EHC = ear and hearing care

**Table 3.** Summary of potential solutions and strategies raised by stakeholders to improve access to EHC

Parameter	Details
Raising awareness campaigns	– Outreach activities, such as hearing screening, with district health centres or local pagoda to engage the community on ear health issues. – Educational community engagement activities including those to help reduce stigma & dispel myths, such as producing visual information (e.g. posters & leaflets) to increase visibility of EHC programmes & local services available & running World Hearing Day events. – Population based prevalence studies to draw attention to the degree & impact of ear disease & hearing loss
Strengthening the EHC workforce	Primary & community-based care
	– Training of nurses in otoscopy within district healthcare centres in consultation with operational districts & the ministry of health at the provincial level; supply health centres with otoscopes. – Educate community healthcare workers in raising community awareness about services available & ear conditions
	Secondary care
	– Training nurses to become audiology technicians (e.g. basic ear diagnosis & treatment, wax removal, hearing testing, hearing aid provision). – Supply equipment to district hospitals (e.g. otoscopes & hearing screening equipment & provide training/monitoring). – Improve quality of services by investing in continuing professional development activities in co-operation with the Cambodian ENT association & international agencies
	Tertiary care
	– Invest in surgical training & education of ENT residents & providers in consultation with senior surgeons; fund or find donor support for surgical equipment (e.g. endoscopes & microscopes). – Incentivise ENT professionals to work in rural areas. – Develop a local tertiary course in clinical audiology
Improving access pathways & co-ordination of care	– Develop stronger referral mechanisms through primary healthcare networks (e.g. educate primary care practitioners on referral pathways). – Inter-organisational co-ordination of care across all sectors (e.g. develop linkages between ENT departments, district hospitals or private hearing care clinics, & district healthcare centres using clear reciprocal referral pathways). – Develop a joint school screening package for hearing & vision through the department of health school health department
Improving government prioritisation & financing of EHC	– Clearer policy on EHC service provision, improve planning to support & improve quality of service provision. – Regulation & governance through clear policy guidelines & professional standards of care. – Develop hearing conversation programmes focused on factory workers, noise assessments due to the lack of prevention & protection measures. – Tax exemptions, or waiving import duties, to improve affordability of hearing aids or equipment. – Subsidise direct & indirect expenses through already established pathways (e.g. Health Equity Fund) for people with chronic ear disease or hearing loss, or children with hearing loss (e.g. subsidise hearing aids, especially for children at Krousa Thmey deaf schools)

EHC = ear and hearing care

### Approachability or ability to perceive

In Cambodia, ear disease and hearing loss are widespread, community awareness is low and access to hearing services in remote or rural villages is a significant issue:

The parents will think that [ear discharge] is normal… so, they will keep the child with the ear discharge since they was born, [until adulthood]. (Provider, public hospital)

Efforts to promote ear and hearing care service providers through targeted outreach activities were variably successful:

We did a lot of school surveys, and it was not very productive. A lot of kids with ear wax and stuff, but we really didn't pick up very many people with acute ear disease, even chronic disease. The problem was those kids were probably not going to school. (Director, non-governmental organisation)

People are generally unaware of ear and hearing care services that are available and seek traditional remedies for chronic disease:

One occasionally encounters outlandish stories, of troubled ears being jabbed with chicken quills or packed with betel and millipede juice. Injured ears being made worse and sometimes damaged irreparably. (Provider, non-governmental organisation)

When people attend formal care, primary healthcare providers often lack competence in the diagnosis, treatment or indications for referral of ear disease and often misinform their patients:

When they come to the hospital, we [found] some doctors didn't advise the parent how to look after the child after they have the ear discharge or ear perforation. (Provider, public hospital)

There's no real treatment for them in Cambodia; the medical system has ignored it… it's officially been what you would call a neglected disease. (Director, non-governmental organisation)

### Acceptability or ability to seek care

Stigma and marginalisation because of low social acceptance of hearing loss or hearing aids are common problems in Khmer society. Children with hearing loss or deafness are commonly hidden from their communities and pushed into manual labour, for example on family-owned farms:

People with [hearing] disability are at home; they are not in society, so we do not see a demand. But when [these] people come to society, the government will see a high demand for this kind of service. (Teacher, special education)

Many hearing children here in Cambodia are still out of school, and some families say, ‘Before I send my deaf child to school, why not my hearing child first, because you know they may have a better future. (Director, disability organisation)

The deaf community, deafness educators and the department of education share opposing views on a nationally recognised mode of sign language (an organic form of Khmer sign language), compared with a signed version of Khmer language. Deafness education is not prioritised, with little buy-in from the government, also evidenced by a lack of sign language interpreters.

Many people are indifferent about their hearing healthcare needs and often display stoicism through a willingness to endure ear pain or discharge, which delays seeking healthcare. These views may be perpetuated by family decision-makers, encouraging easily accessible traditional remedies or over-the-counter pharmaceuticals over care in the formal healthcare system, compounding delay in seeking effective treatment and reinforcing ‘health shopping’ behaviour. Barriers related to fear of doctors, surgical procedures, anaesthesia and ill-treatment or exploitation at modern health facilities were reported:

Ninety per cent of people have tried something [else] before: pharmacy, traditional medicine, health centre, local clinic, government hospital, NGO [non-governmental organisation]. But remember, patient like to walk around and [then] decide where they want to get treatment. It can be about trust. (Provider, non-governmental organisation)

### Availability or ability to reach

There is a lack of ear and hearing care workforce cadres, such as primary healthcare workers trained in primary ear and hearing care, audiologists, speech pathologists, deaf education specialists, sign language interpreters or ENT surgeons with advanced otological surgical skills training, especially in rural areas. A non-governmental organisation provides otology, audiology and related sciences training to staff members, working in partnership with several aid organisations and public hospitals across the country. A non-governmental organisation hospital has trained several ENT nurses in otoscopy, ear toileting, hearing screening, diagnostic testing and aural rehabilitation and they take on task-sharing responsibilities. Increasing numbers of ENT specialists have graduated from tertiary institutions. However, few are sufficiently trained in advanced surgery for chronic ear disease, and this is compounded by a lack of appropriate surgical equipment or training facilities:

There are few surgeons who are able to treat the ear problem or provide the surgery. We still have a poor resource, human resource, to diagnose the hearing loss and fit hearing aid. (Provider, non-governmental organisation)

Assessment of hearing status of infants or newborns is limited to a few providers trained in diagnostic evoked potential tests, and only two public hospitals in Phnom Penh have access to evoked potential machines. A non-governmental organisation hospital in Kampot provides screening of newborns using otoacoustic emissions, but those referred for further diagnostic evoked potential assessment need to travel to the city. This is an issue given cultural beliefs that mothers and newborns should remain indoors for the first three months post-delivery to keep safe and warm. There is also a critical shortage of audiological equipment, facilities (including sound-treated rooms), ear mould making facilities and hearing aid verification machines. As a provider from a public hospital said, there are:

*…* low skill service providers and lack of equipment may limit them … [we have] no instrument [for] audiometry, no financial support, and poor hearing company available. (Provider, public hospital)

The provision of hearing aids in Cambodia is fragmented with no viable supply chain, local manufacturer representation or repair facilities. The issue is also compounded by poor user experience with low-quality and unreliable hearing devices, a problem found across many low- and middle-income countries.^37^ Facilities providing hearing aids are heavily reliant on donated devices from international agencies:

Before, we could make an [ear]mould, or we had hearing aids from French mission or donations… but now we cannot do these activities [because] the donations and activities stop for a while. (Provider, non-governmental organisation)

Despite the commitment of the ministry of health in strengthening governance and quality of care, ‘the public health system remains limited in its ability to provide high-quality, and high-volume healthcare’ (provider, non-governmental organisation). Hospital ENT departments across all sectors are under strain because of high demand, long wait times and insufficient time during consultations to provide optimum clinical care:

So, my problem right now in my department, we have [many] patients but we don't have enough staff. We have a lot of people: nurse, but they have multifunction and [too] few doctors, so we can't provide all the things quick to what the patient need. (Provider, non-governmental organisation)

District referral hospitals delivering the complementary package of activities do not have ENT departments, so patients with chronic disease cannot access specialist care and instead enter into a cycle of medical review. Key informants believe this is a failing of the public health system in which ear and hearing care is neither understood nor prioritised.

I don't think our health public health service is capable enough to really detect hearing of people who have hearing loss… or whether we do not have the facilities, or we just don't care… [The] main obstacles [why] the service is very limited: it's because of capacity, it's because of [lack of] investment, and it's because of a lack of awareness. (Director, disability organisation)

International volunteers provide mentorship, training, resources and support through short-duration visits to hospital ENT departments. However, several providers were frustrated by their lack of local training opportunities and over-reliance on visiting foreigners.

### Affordability or ability to pay

People with ear disease face significant financial and opportunity costs and time away from work, especially those from rural regions where services are lacking:

People feel like it's too far away… and for poor people they don't have the money to come here, and some people, they have money, but they don't have time because they had to work. (Provider, non-governmental organisation)

Across all sectors, high user fees for screening, diagnostic assessment, hospital fees, treatment, surgery, anaesthesia and medication preclude care-seeking, and patients try alternative treatments. When symptoms persist or worsen, care is sought at pharmacies, in the public sector (local health centres, referral hospitals or district hospitals), in the private sector (private clinics, hospitals or non-governmental organisations) or a combination of these. Reducing user fees for public health services through the national poverty indentification system (health equity fund) has increased access to some ENT services:

At [public] hospitals now, we have the service sponsored by the government, ‘Somathore Sokhaphibal [Health Equity Fund]’. So, if they can access and come to the hospital, the government will pay for all the treatment, the surgery, the hospital fees. It just started about one and a half the year [ago] and, from day to day, the number of the patients from rural areas has increased sharply. (Provider, public hospital)

Funded insurance schemes are available for public officials or through social health insurance programmes for the private sector:

… ear care is much assured by National Social Security Fund for sewing factory workers, [athletes] and government staff; whereas very poor people, handicap or disabled, can access healthcare by health equity fund, but the others like farmers have to pay their own money for ear service care. (Provider, public hospital)

At private hospitals or non-governmental organisation facilities, user fees are based on proxy means testing. Some non-governmental organisations and international agencies provide free services, or cover transportation, treatment, food and accommodation costs for outreach visits.

There are no funding mechanisms for hearing aids, cochlear implants or listening devices, making them prohibitively expensive for most Cambodians. Some providers offer used hearing aids from international donors for purchase or rent. However, most cost $200–300 for a basic analogue trimmer hearing aid, or over $600 for a digitally programmable device. Cochlear implants cost $13 000, which includes assessment, surgery, mapping, rehabilitation and speech therapy for two years post-surgery. However, as payment is usually up front, access to such technology is limited to wealthy families or those willing to risk potentially catastrophic expenditure.

Other financial costs include transportation, accommodation, and direct or indirect medical or rehabilitation expenses. Users assemble finances to pay, borrow from family or friends, sell assets, or access savings. People with untreated hearing loss or deafness face further disadvantage through opportunity costs, including the potential loss of earnings:

When we go to the field, we learned that deaf people are among the poorest population in Cambodia, so they are not able to pay. (Provider, non-governmental organisation)

### Appropriateness or ability to engage

Hospital providers often focus on ensuring they meet their quota for patients or surgical procedures, with little mention of the provider–patient relationship. Patient-centredness in working with patients with hearing impairment is an ongoing challenge:

[Our staff need] a mind to work with the patient who has the ear problem. It's like the same thing every day. It takes time about the cleaning the ear, about the work with the children who has hearing loss, about the work with the patient who is deaf. We have to shout; we have to talk nice, so it's not easy work. (Provider, non-governmental organisation)

Weak linkages between providers result in little collaboration or co-ordination of care. Some informal linkages and referral networks have developed between organisations, but most providers across the health system work independently and rarely cross-refer, in some instances to retain their income stream. Consequently, patients with chronic ear disease often fall into a recurrent review cycle, bearing significant direct and indirect out-of-pocket expenses:

Some doctors, or some centres doesn't want to transfer the patient to us because they are afraid to lose their patient. They may lose face because they can't perform the surgery [if] they have no training [or] no equipment. (Provider, non-governmental organisation)

Notably, rivalry between some service providers in the ear and hearing care sector is common:

… you have the competition between the NGO [non-governmental organisation], the community, the government, the people and the hospitals. You have several hospitals in competition, [so it's] not always very clear about co-operation. (Provider, non-governmental organisation)

Previous attempts at inter-organisational collaboration between multiple stakeholders from private and public facilities for priority planning of future services failed because of competing views and misalignment of priorities.

We rarely see [providers] come together because they have different [opinions] … for children with hearing loss [or deafness], [we] assess the way they need education, but the medical [sector], they see the children with the need to treat and recover it. (Teacher, special education)

Respondents described limited government awareness and responsiveness to ear and hearing care needs. Government ministries work as separate entities, with limited interaction, co-operation and clarity of their role in ear and hearing care service delivery. Non-governmental organisation hospitals reporting their ear and hearing care activities to the ministry of health described little interest, support or direction from the government:

We inform the Ministry of Health, but they are not interested about what we are doing. We don't know because we never receive any [response]. (Provider, non-governmental organisation)

… each director of their ENT service is his own boss [with] no directive from the Ministry of Health … the people working in health departments [should] share a common target, and I don't think this is the case. (Provider, non-governmental organisation)

### Potential strategies and solutions

Stakeholders discussed potential strategies and solutions to improving access to ear and hearing care services, summarised under four thematic areas (Table 3). Stakeholders described the importance of collectively listening to local needs, raising community and government awareness to address negative perceptions and stigma, and promoting ear and hearing care prioritisation. Other strategies included strengthening the ear and hearing care workforce through targeted training at the community, primary, secondary and tertiary levels of care and improving access pathways through a more robust inter-organisational referral mechanism, linking primary care with other levels of care. Finally, several key considerations included solutions aimed at improving government prioritisation and financing for ear and hearing care services, developing more explicit policy guidelines and professional standards of care, improving the delivery of care, and linkages between the ministry of education and school health department programmes.

## Discussion

The exploration of key informants’ perceptions and experiences through the lens of the Levesque framework provides a unique snapshot of the status of ear and hearing care in Cambodia. Key informants were enthusiastic about relaying their experiences, and some shared the significant developments where the public could access ear and hearing care services at the National Hospital through social protection mechanisms, amongst other accomplishments within the non-governmental organisation sector. On the other hand, a diverse range of supply-side challenges were overwhelmingly described. These included low community awareness of the services offered; shortages of ear and hearing care workforce cadres, especially in rural areas; limited training opportunities; lack of infrastructure, supply-chain and resource shortages supporting early diagnosis and early intervention; high direct, indirect and opportunity costs; and a lack of co-ordination of ear and hearing care services within the health system. On the demand side, low community literacy, stigmatisation, geographical barriers, low earnings and attitudes toward healthcare shopping or lack of belief in medical providers influenced the uptake of services. The urban location of services may also lead to the restricted spread of information.

Notwithstanding the wide range of barriers, which speak to different service delivery components, two overarching points emerge from the thematic analysis. First, the most pressing issue is the lack of ear and hearing care providers across the country, especially in primary care facilities in rural areas, which means a significant number of Cambodians suffering preventable and treatable ear conditions do not have access to ear and hearing care services. The introduction of the health equity fund has stimulated public ear and hearing care service utilisation at the national hospital level for holders of the IDPoor card. Yet, the vast majority of district hospitals or health centres have limited capacity to cater for people with ear or hearing conditions. Cambodians continue to rely heavily on private sector services at pharmacies or clinics that remain largely unregulated, enduring high out-of-pocket costs.^38^ Low-quality ear and hearing care services subject patients to unnecessary repetitive and expensive treatments, potentially pushing them further into poverty.^39^

Second, many of the accessibility challenges identified in this study echo those found in other low- and middle-income countries, which confirms the uniformity of these accounts in other contexts.^10^ These include ear and hearing care workforce shortages,^40,41^ inadequate referral systems and limited communication between providers,^42,43^ lack of aural rehabilitation,^44,45^ low awareness or knowledge of ear and hearing care services amongst other healthcare providers and the community,^46,47^ and high demand leading to long wait times.^42,48^ However, given the qualitative nature of this work, our results cannot be generalised across the country, and further quantitative research to verify the scale of these barriers is required at regional and national levels.

Ear and hearing care services are not a mainstream component of the Cambodian healthcare system, as evidenced by a lack of national planning and current infrastructural capacity to meet the need across the private and public sectors. Stakeholders described a counterproductive inter-professional culture that points to a fragmented, unco-ordinated and highly variable delivery of ear and hearing care services. These findings substantiate and extend our previous study on patient experiences at a non-governmental organisation tertiary hospital in Phnom Penh,^4^ where patients endured chronic symptoms for an average of 13–14 years before reaching formal care. Ineffective referral networks hinder access to treatment and contribute to high morbidity of ear disease; however, fostering interdisciplinary relationships across institutional boundaries can build the trust required for co-ordinated service planning and development.

In the last 20 years, alongside significant economic growth, Cambodia has seen substantial improvements in health outcomes, life expectancy, reduced maternal mortality and improved child health and nutrition.^21,25,49,50^ The introduction of social health protection mechanisms, such as the health equity fund and others, have significantly reduced out-of-pocket user fees, supporting equity in access to adequate and affordable health services across the country.^15^ Unaddressed hearing loss, however, is a silent burden, costly not only to the individual but also to the economy.^51^ According to the Cambodia Demographic and Health Survey (2014), 3 per cent of the general population over 5 years old has hearing issues,^52^ but no other data on hearing loss or ear disease exist, making it difficult to estimate the economic costs associated with the unmet need. The global estimated economic costs incurred from healthcare, educational support, loss in productivity and quality of life costs related to hearing loss exceed $981 billion.^53^ Epidemiological modelling suggests that these costs can be reduced through public health interventions incorporated into a national strategy.^3^

Now is an opportune time to look at the national strategy to build on the successes of the ministry of health in its notable response to the increasing burden of non-communicable diseases through targeted, integrated care.^54^ The Cambodian health system is strategically well placed to consider formal pathways to bring ear and hearing care under the national healthcare policy and strengthen the capacity of healthcare facilities and hospitals to better cater to the country's hearing healthcare needs.^3,4^ Patients would benefit from the social health protection mechanisms already in place without reallocating funding arrangements.

The lack of ear and hearing care workforce representation at the primary care level is of particular concern, as this is the most common entry point into the public healthcare system for the population. Strengthening the ear and hearing care workforce across all geographical regions through primary healthcare and community-based care models to reach underserved populations has been recognised in other contexts.^46,55^ Within district healthcare centres, expanding the minimum package of activities to include primary ear and hearing care and community-based hearing rehabilitation could drive people away from less expensive non-health providers.^56,57^ In Mozambique, community health workers play a significant intermediary role between the health system and community, facilitating referrals to ensure a continuum of quality primary care.^58^ Community health workers can take an advocacy role in community education to increase ear and hearing care literacy, increase awareness of the role and location of ear and hearing care providers, dispel myths and reduce stigma. Investment in mobile health technologies can also support task-shifting or task-sharing initiatives whereby community health workers can train in screening for ear disease or hearing loss.^59^ More research is required to understand how community health workers can provide hearing aids and other assistive devices and how primary ear and hearing care can be integrated without sacrificing the existing quality of care.^55^

- Despite the reported high prevalence of ear disease in South-East Asia, few studies have explored access to care for those with ear and hearing conditions
- Significant improvements in health outcomes have been observed in Cambodia since the introduction of health financing policies to improve health service delivery amongst the most vulnerable
- There are numerous challenges, such as the lack of infrastructure, shortage of workforce in primary care and rural areas, poor supply chain and delivery of assistive products and services, and poor co-ordination of services
- These barriers limit access to care for the many Cambodians suffering from preventable and treatable ear conditions
- Now is an opportune time to examine the national healthcare strategy to build on the success of the ministry of health
- Adopting such policies can allow patients to access the current social protection mechanisms to better cater to the country's hearing healthcare needs

A broader analysis of healthcare providers offering ear and hearing care through the complementary package of activities at district referral hospitals would provide helpful information on how the scope of primary healthcare can be broadened to detect, treat or refer patients with ear conditions earlier. For successful integration, resources such as the WHO toolkit can aid these services’ development, implementation and scaling up.^60^ Linking ear and hearing conditions and other health priorities may also promote ear and hearing care services between health professionals and encourage interdisciplinary collaboration.^61^

In line with its commitment to the Incheon strategy for the Asia Pacific,^13^ which aims to implement a national policy to improve the provision, quality and efficiency of rehabilitation services at the community level,^14^ there is a critical need for investment to improve access and affordability of assistive hearing devices. Access to hearing aids is a huge challenge, especially in South-East Asia where only 16 per cent of people in need of a hearing aid have access to one.^62^ Many of the challenges, including the high cost, lack of human resources and services, and lack of awareness of the benefits or associated stigma have been previously reported.^63,64^ The delivery of alternative service delivery models utilising low cost technologies that do not rely on trained professionals, alongside appropriate policy and regulatory changes, could facilitate access.^62^ Surveying and interviewing aural rehabilitation product suppliers would provide vital information to shape interventions for improving the supply and coverage of hearing aids and other listening devices.^65^ The use of role models who experience hearing loss or deafness, promoting benefits of hearing aids or sign language and including such people in policy dialogues, could help address stigma and empower people with hearing loss.^3^

### Limitations

Given the coronavirus disease 2019 restrictions, face-to-face communication was not always possible, and several interviews or questionnaires were conducted remotely, which may have restricted open discussion. This research is not a quality assessment of ear and hearing care service provision; it seeks to understand key informant perceptions of the accessibility and quality of the services available. As participants were purposefully selected, we acknowledge that views are not representative of all stakeholders working in ear and hearing care in Cambodia, so the findings cannot be generalised to other contexts. Despite these limitations, this study highlights essential questions in obtaining equitable access to ear and hearing care in Cambodia, which may apply to other low- and middle-income countries. Population-based quantitative data could be used to further analyse potential challenges and reinforce these findings. The local burden of disease data would also spur interest in the economic costs of untreated ear disease and direct policymakers towards policies that strategically forecast primary health, surgical and rehabilitation workforce needs. Prevalence data can also help monitor the impact of future interventions and advocate for increased resources.^66^ Further studies on out-of-pocket payments for ear and hearing care services from a broader range of providers would also better represent direct expenses.

## Conclusion

This study has shown there are inequities in access to ear and hearing care in the Cambodian context, with the health system primed to meet the needs of the wealthy, missing those living in poverty or geographically distant areas or marginalised conditions. Stakeholders described a lack of ear and hearing care services to meet the demand, especially outside Phnom Penh in primary care and aural rehabilitation. Supply-side challenges include a shortage of trained ear and hearing care professionals, facilities and resources, poor co-ordination between providers, non-existent referral pathways and long wait times. Now is an opportune time to investigate and build on the positive trend of integrated care for non-communicable diseases within the health system. Ear and hearing care can be positioned strategically within primary care to strengthen the minimum and complementary package of activities delivered through district health clinics and referral hospitals. This would build on the effectiveness and responsiveness of the health system to the population's hearing healthcare needs, while assuring affordability through the financial protection mechanisms in place.

## Supporting information

Waterworth et al. supplementary material Waterworth et al. supplementary material

## Supplementary material

The supplementary material for this article can be found at https://doi.org/10.1017/S0022215122002158.
