# Supplementary material for: Access to ear and hearing care services in Cambodia: a qualitative enquiry into experiences of key informants
Source: J Laryngol Otol. 2022 Sep 26;138(1):22–32. doi: 10.1017/S0022215122002158 (PMC10772024; doi:10.1017/S0022215122002158)
Supplement: Waterworth et al. supplementary material [file S0022215122002158sup001.docx]

<JLO 22-0540; supplementary material>

<Ahead>

**Questionnaire**

<text>

<Ahead>

**Introduction**

<text>

You have been invited to participate in this research as a provider of ear care services, to find out more about your health service and the barriers to providing ear and hearing care that you see in Cambodia. Please answer the following questions as best you can. Your answers are confidential, and responses are anonymous. Please ensure you have read the plain language statement and signed the consent form which indicates that you agree for your responses to be used for research.

**Table 1.** Supply for ear care services

| 1. Firstly, please tell us about yourself and your experience with ear care (no identifying information will be retained or used). Tell us about the services you provide. |
| --- |
| 1. Do you offer screening/outreach and information? |
| 1. In what locations? With what facilities? With what technical inputs? Are services readily available? |
| 1. Are you satisfied with the equipment you have access to here? |
| 1. Is ear care seen as a high priority by the organisation? |
| 1. Tell me about service availability at a national level for ear care? |
| 1. How does ear and hearing care compare to other healthcare priorities by the government? |

| Demand for ear care services |
| --- |
| 1. Tell me your opinion about the need (demand) for ear care in Cambodia. |
| 1. What type of ear conditions do you see amongst those who use your services? |
| 1. What do you see are the common determinants (or causes) of hearing loss in this country? |
| 1. Do you think the need is currently being met? |
| 1. Do you see access to ear care as an issue for people in Cambodia? If so, how? |
| 1. What do you see as the main issues? |
| 1. Who misses out on the services? Which groups of people? |
| 1. Reasons and examples for certain groups who might miss out accessing services? |

| Approachability (visibility of service) |
| --- |
| 1. Tell me about how people find ear services. Have you experienced difficulties trying to reach people? |
| 1. How do you get information about your services to the general public? How does this compare to how other health services? (e.g. eye care) |
| 1. If you offer hearing screening: tell me about any challenges you see for people coming to the screening services? Are there challenges for organising screening services in the community? |

| Acceptablity |
| --- |
| 1. Can you tell me about people’s beliefs about their ear problems? Do their beliefs affect their openness to seeking help for their ear problems? |
| 1. Do you think the current services are acceptable to patients? If not, why not? |
| 1. How do people view hearing problems and ear disease in Cambodia? Do people experience stigma? If so, does this affect their willingness to seek care? |
| 1. Do you think people’s beliefs about their ear problems stop them from accessing or accepting ear care services? |
| 1. Are there cultural or social reasons why people may not wish to attend for ear care? (e.g. gender/social status) |
| 1. Do you think people are generally trusting or fearful of coming for health services? How about ear care? |

| Availability/accessibility |
| --- |
| 1. Do you think patients know about ear care services they can access? |
| 1. Is it easy to get to appointments? Is there any waiting time? |
| 1. Are there any reasons (characteristics) that might stop people from attending? (e.g. distance, hospital). |
| 1. Are people able to access transportation and the building adequately? |
| 1. Do you offer after-hours service? |

| Affordability |
| --- |
| 1. Tell me about what costs there are for people to receive care for their ear problems in Cambodia. Do you think ear care is affordable to people who need it in Cambodia? |
| 1. How are costs for ear care usually met? (e.g. Do they have to borrow money? Are they able to access the health equity fund?) |

| Appropriateness |
| --- |
| 1. Tell me about the training and ability of staff to provide ear care services in Cambodia. |
| 1. Do you see a lot of variation (differences) in the quality of care between different providers? |
| 1. Do you have many people who attend but do not accepting the recommended treatment? Can you provide any examples? |
| 1. Does the service meet the need? |

| Conclusion |
| --- |
| 1. If I could give you a magic wand, what are your hopes and dreams for providing ear care services in the future for this country? |
| 1. How do you see the role of ear and hearing care changing in the future? |
| 1. How do you see primary healthcare working in future for ear related services? |
| 1. Is there anything you would like us to know? |

<Ahead>

**Topic guide**

<text>

<Ahead>

**Introduction**

<text>

Spend time building rapport with participant.

Remind the participant that this will be audio-recorded and translated into English (then switch on recorders).

<np>Today, I am interested in finding out more about your health service, in particular, what the barriers to ear and hearing care that you see here, in Cambodia.

<Ahead>

**Background (about respondent)**

<text>

Firstly, tell me a bit about yourself and your experience with ear care (no identifying information will be retained or used). Tell me about the services you provide.

<np>Prompts:

- Do you offer screening/outreach and information?
- In what locations? With what facilities? With what technical inputs? Are services readily available?
- Are you satisfied with the equipment you have access to here?
- Is ear care seen as a high priority by the organisation?
- Tell me about service availability at a national level for ear care? How does ear and hearing care compare to other healthcare priorities for the government?

<np>Tell me your opinion about the need (demand) for ear care in Cambodia.

What type of ear conditions do you see amongst those who use your services?

<np>Prompts

- What do you see are the common determinants (or causes) of hearing loss in this country?
- Do you think the need is currently being met?
- Do you see access to ear care as an issue for people in Cambodia? If so, how? What do you see as the main issues?
- Who misses out on the services? Which groups of people? Reasons and examples for certain groups who might miss out on accessing services?

<np>I’d like to spend some time talking about various barriers that you might experience in relation to providing health services, and I wonder whether these also apply for ear-related health services.

<Ahead>

**Approachability (visibility of service)**

<text>

Tell me about how people find ear services. Have you experienced difficulties trying to reach people?

<np>Prompts:

- How do you get information about your services to the general public? How does this compare to other health services? (e.g. eye care)
- If they offer hearing screening: tell me about any challenges you see for people coming to the screening services? Are there challenges for organising screening services in the community?

<Ahead>

**Acceptablity** **(cultural and social factors)**

<text>

Can you tell me about people’s beliefs about their ear problems? Do their beliefs affect their openness to seeking help for their ear problems? Do you think the current services are acceptable to patients? If not, why not?

<np>Prompts:

- How do people view hearing problems and ear disease in Cambodia? Do people experience stigma?

If so, does this affect their willingness to seek care?

- Do you think people’s beliefs about their ear problems stop them from accessing or accepting ear care services?
- Are there cultural or social reasons why people may not wish to attend for ear care? (e.g. gender/social status)
- Do you think people are generally trusting or fearful of coming for health services? How about ear care?

<Ahead>

**Availability/accessibility** **(capacity of service to be reached or provide service)**

<text>

Do you think patients know about ear care services they can access?

<np>Prompts:

- Is it easy to get to appointments? Is there any waiting time?
- How long does it take for people to access ear care from the time they had the problem, if they know they have a problem?
- Are there any reasons (characteristics) that might stop people from attending? (e.g. distance, hospital).
- Are people able to access transportation and the building adequately?
- Do you offer after-hours service?

<Ahead>

**Affordability**

<text>

Tell me about what costs there are for people to receive care for their ear problems in Cambodia. Do you think ear care is affordable to people who need it in Cambodia?

<np>Prompts:

- How are costs for ear care usually met? (e.g. Do they have to borrow money? Are they able to access the health equity fund?
  - Direct costs may include: surgery, hearing aid/batteries, transport, food, clothes, accommodation, emergency care, informal payments (e.g. cost of childcare)
  - Indirect costs (e.g. do they give up work/loss of earnings during surgery/recovery).
  - Loss of opportunity costs (give up work).

<Ahead>

**Appropriateness (technical and interpersonal skills, timeliness, quality of care)**

<text>

Tell me about the training and ability of staff to provide ear care services in Cambodia.

<np>Prompts:

- Do you see a lot of variation (differences) in the quality of care between different providers?
- Do you have many people who attend but do not accept the recommended treatment? Can you provide any examples?
- Does the service meet the need?

<Ahead>

**Conclusion**

<text>

If I could give you a magic wand, what are your hopes and dreams for providing ear care services in the future for this country?

<np>Prompts:

- How do you see the role of ear and hearing care changing in the future?
- How do you see primary healthcare working in future for ear related services?

Is there anything you would like us to know?
